# Epidemiology and survival of cervical cancer in Iran based on national cancer registry data (2008-2014)

**DOI:** 10.3389/fonc.2023.1132369

**Published:** 2023-04-19

**Authors:** Atieh Akbari, Maryam Khayamzadeh, Reza Salmanian, Afshin Moradi, Mohammad Esmaeil Akbari

**Affiliations:** ^1^ Cancer Research Center, Shahid Beheshti University of Medical Sciences, Tehran, Iran; ^2^ Academy of Medical Sciences, Tehran, Iran

**Keywords:** epidemiology, incidence, Iran, survival, uterine cervical neoplasm

## Abstract

**Background:**

Cervical cancer (CC) is the third most commonly diagnosed cancer and the fourth leading cause of cancer death in females worldwide, associated with the incidence of human papillomavirus (HPV) infection. The CC incidence is low in Iran, ranking 11th among cancers. This study aimed to estimate the survival rate of CC and the reasons for its low survival rate based on the data retrieved from the Iranian National Cancer Registry System.

**Methods:**

In this retrospective cohort study, data for patients diagnosed with CC from 2008 to 2014 were collected and analyzed. The Kaplan-Meier method was used for survival analysis based on epidemiological and clinical factors.

**Results:**

A total of 5,304 women were diagnosed from March 10, 2008 to March 9, 2014 and 2,423 patients were followed. The mean age of the cases was 51.91 years, and 65.91% were alive. The 5- and 10-year survival rates were 58% and 50%, respectively, with no difference between younger cases with SCC or AC but better survival rates for older patients with SCC.

**Conclusions:**

As a preventable disease, CC is related to biological factors and geographical and sociodemographic indices. Geographical, cultural, and religious behaviors affect the CC incidence and survival. In Iran, the 5-year survival rate ranges from 34% to 70% among different geographic regions. Hence, effective screening based on cultural and sociodemographic issues is recommended.

## Introduction

Cervical cancer (CC) is the third most commonly diagnosed cancer and the fourth leading cause of cancer death in females worldwide (1). More than 90% of patients with CC show positive human papillomavirus (HPV) infection especially in developed countries (2, 3). The highest prevalence rate of HPV is in Africa, the Caribbean Islands, and South America, while the lowest rate is observed in Australia, New Zealand, and Western Asia (4). Unlike in western countries, in Iran, as in other Muslim countries, HPV prevalence is not high among younger women (1, 5).

The CC incidence in Iran is reported to be less than that of European and American countries, ranking 11th among female cancers (6). According to 2016 assessments, the age-standardized incidence rate of cervical cancer was 2.4 (1.7-3.3) per 100000 women, and the age standardized mortality rate for women with this cancer was 0.98 per 100000 women (7).

Cervical cancer is one of the most important preventable cancers, which can be diagnosed early with screening. After introducing of the Pap smear as a routine screening test for CC, the mortality rates declined by 50-75% over the past 50 years (8). In middle-income countries like Iran, there is no established national screening program, instead there is an “opportunistic screening,” meaning that patients, by their knowledge or visiting by gynecologists or health workers for other reasons are advised to have the test. Opportunistic screening is not usually extensively fully covered, which leads to the diagnosis of patients with CC in advanced stages and, subsequently, lower survival (9, 10).

Despite low CC incidence in Iran and other Muslim countries because of different cultural and behavioral factors compared to western countries, the lower survival rates and higher mortality rates need to be considered (9).

This study aimed to describe the survival rate of CC in various age groups, regional center distribution, and pathological differences based on the Iran National Cancer Registry (INCR) ^13^ from 2008 to 2014 (11).

## Methods

In this retrospective cohort study, patients diagnosed with CC and registered in INCR from March 10, 2008 to March 9, 2014 were included. In the national cancer registry system, all new malignancy cases are registered from all over the country and gathered in the Ministry of Health and Medical Education. This registry system was pathology-based in the years of study, with a coverage rate of approximately 93% of pathological reports. After that, it became population-based, collecting the data of new cancer patients from pathology laboratories, hospitals, and death certificates. We received the data from the cancer office of the Ministry of Health and Medical Education. The data included the demographic and pathologic characteristics of the patients and tumors. All patients diagnosed with ICD10 codes C53 series were extracted from the database. The method of omitting duplicate data was explained in our previous research. First, a technician checked the data and omitted repeated registered cases during the mentioned period (2008–2014) ^14^. Then, some trained nurses followed the patients by calling those whose telephone numbers were available to determine their status (alive or dead) at the end of the study (2019). Some cervical cancer cases that could not be reached by repeated calls were considered as censored cases. Survival was calculated, indicating the duration from the date of diagnosis (pathology report date) to the date of death or last follow-up. The participants were categorized into 16 age groups with a 5-year interval to show their age distribution, and they were divided into four groups (<30, 30-44, 45-59, and ≥60 years) for survival analysis. The pathologic types of the tumor were registered in different groups. The participants were divided into four main groups: Squamous Cell Carcinoma (SCC), Adenocarcinoma (AC), other primary epithelial cell carcinomas, and non-epithelial cell carcinoma. Iran contains 31 provinces. Because of the small numbers of cervical cancer patients in some provinces due to their smaller populations, we decided to use the categorization recommended by the Ministry of Health and Medical Education, which divides the country into 13 regions based on socioeconomic statuses such as culture, the local language, and geographical regions. The data were described by the mean, standard deviation (SD), number, and percentage. The Kaplan-Meier method was used for survival analysis, and the log-rank test was used to show the difference between the survival of different groups. The Cox proportional-hazards regression model was used to assess the effect of patient characteristics on survival rate. D The data were analyzed using SPSS version 24, and *P*-values less than 0.05 were considered statistically significant.

This study was conducted following the Declaration of Helsinki and approved by the Shahid Beheshti University of Medical Sciences Ethics Committee (reference number: IR.SBMU.CRC.REC.1398.029)

## Results

The demographic and tumor characteristics of the primary CC diagnosed from March 10, 2008, to March 9, 2014 and obtained from the INCR system were summarized and analyzed. During these years, 5304 females with CC were registered. After excluding 156 duplicate cases, with constant geographical distribution, 2423 patients were followed (the success rate of follow-up was 45%). The mean ± SD age at the diagnosis was 51.91 ± 13.97 years (range: 10-97). Based on INCR, CC cases increased from 556 patients in 2008 to 671 patients in 2014.

As shown in **Table 1**, after dividing Iran into 13 regional centers, the lowest mean age was 50.55 years in Khorasan and 50.98 in Tehran, whereas the highest mean age was 55.26 years in Guilan.

**Table 1:** The mean age of patients with cervical cancer in regional centers.

Regional center	Mean age (SD) (Year)
Hamedan	52.77 (13.40)
Fars	51.59 (13.32)
Tehran	50.98 (13.82)
Khorasan	50.55 (13.18)
Kerman	51.92 (13.48)
Alborz	51.74 (13.17)
Kermanshah	52.66 (13.41)
Qom	53.80 (13.88)
Azerbaijan	52.27 (13.45)
Khuzestan	52.69 (13.54)
Isfahan	54.18 (13.50)
Mazandaran	51.59 (13.40)
Guilan	55.26 (13.64)

**Figure 1:**
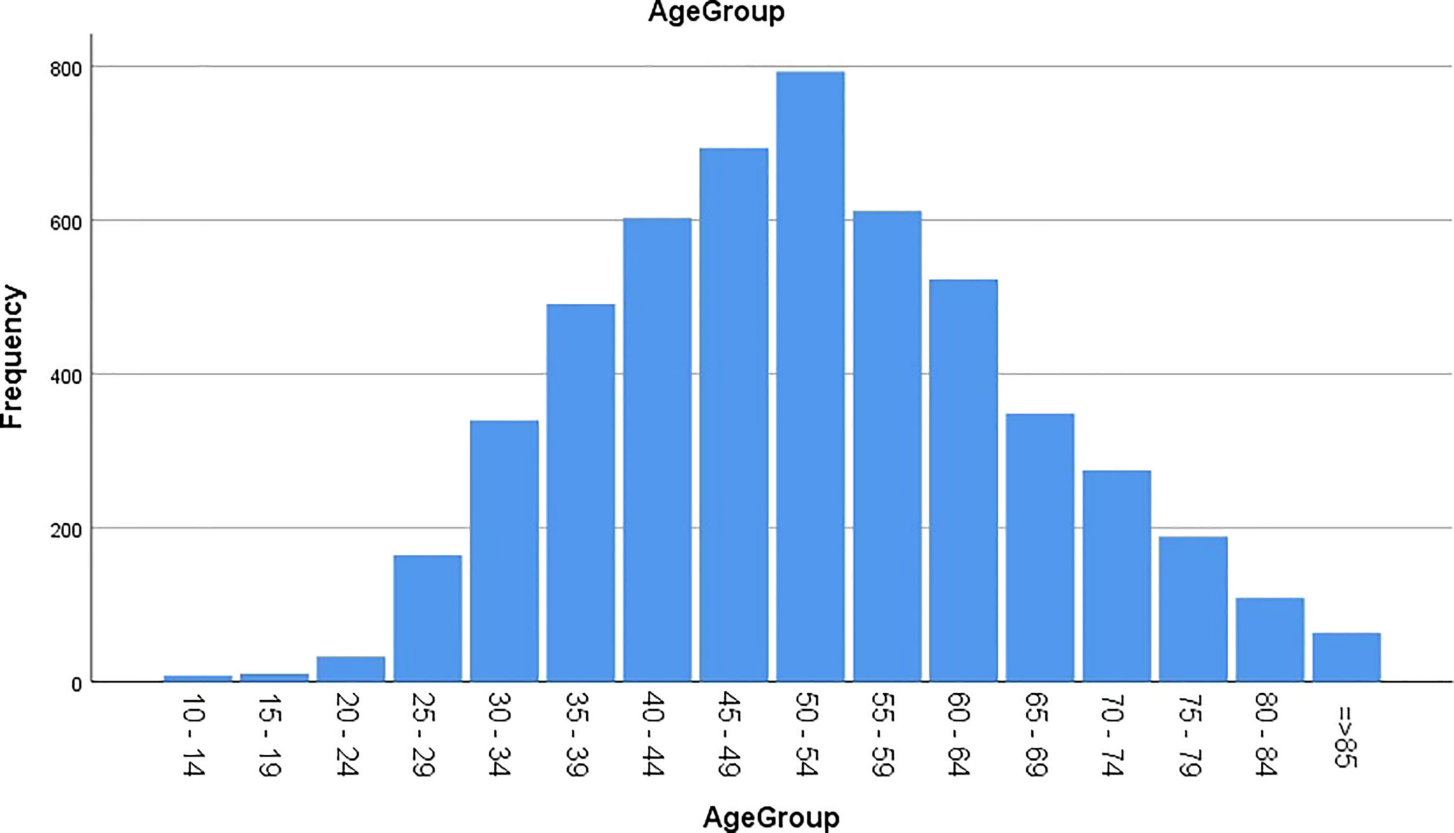
Age distribution of patients with cervical cancer in Iran during 2008-2014.

The age groups were categorized from 10 years or greater to 85 years or more by five-year intervals (**Figure 1**). The highest proportion of the disease was reported in the age group of 50 to 54 years (15.16%). Most of the patients were distributed from 45 to 54 years (28.27%), and 10.06% of the CC patients were 30 years old or younger at diagnosis.

**Figure 2:**
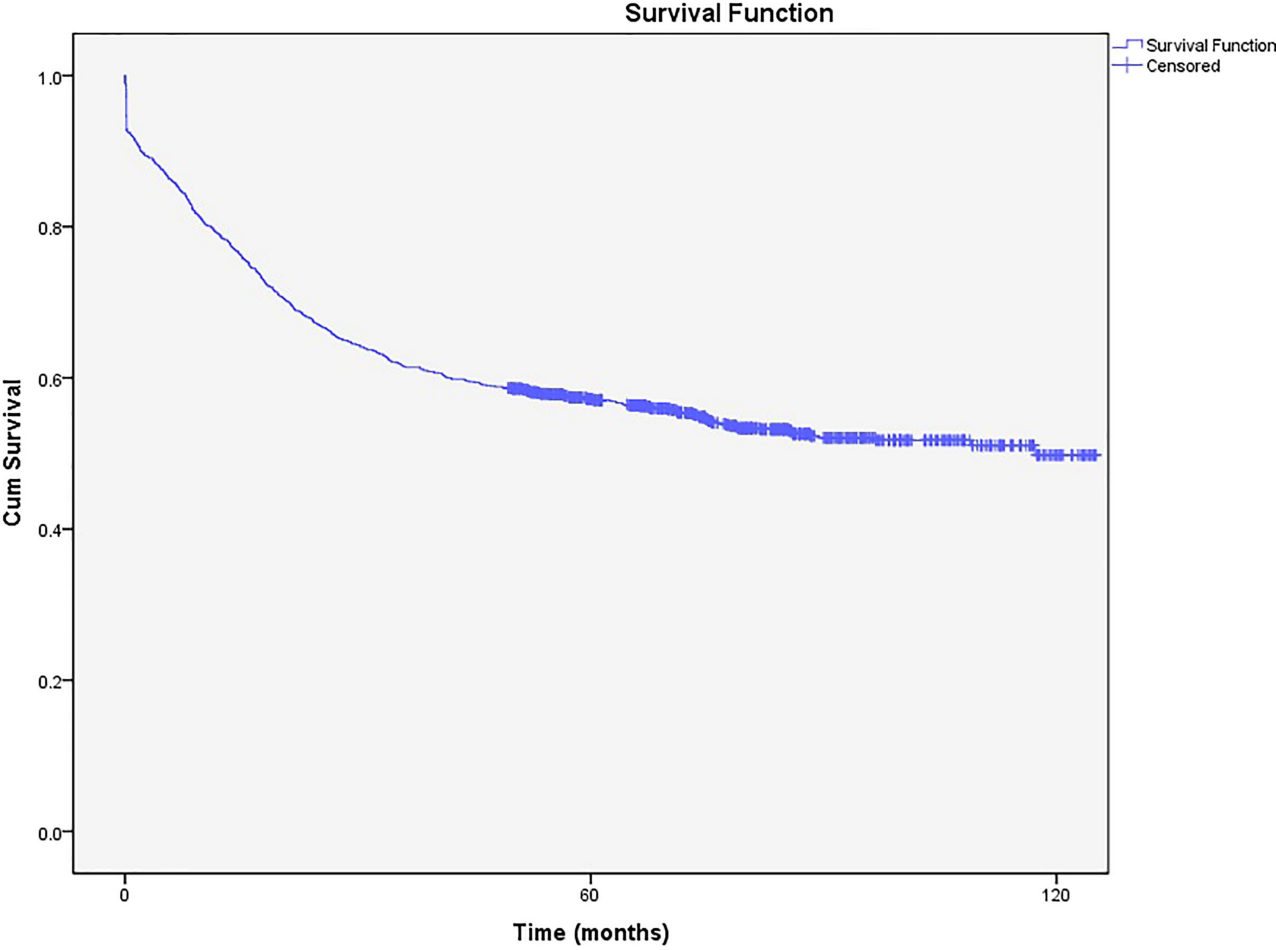
The 5- and 10-year survival rates of cervical cancer from 2008 to 2014.

Of the patients followed in the present study, 826 (34.08%) were dead, and the rest were alive (n=1597, 65.92%). According to the Kaplan-Meier curve, the 5-year survival rate was 58% (57.98%- 58.0.02%), while the 10-year survival rate was 50% (49.96%-50.04%) (**Figure 2**).

**Figure 3:**
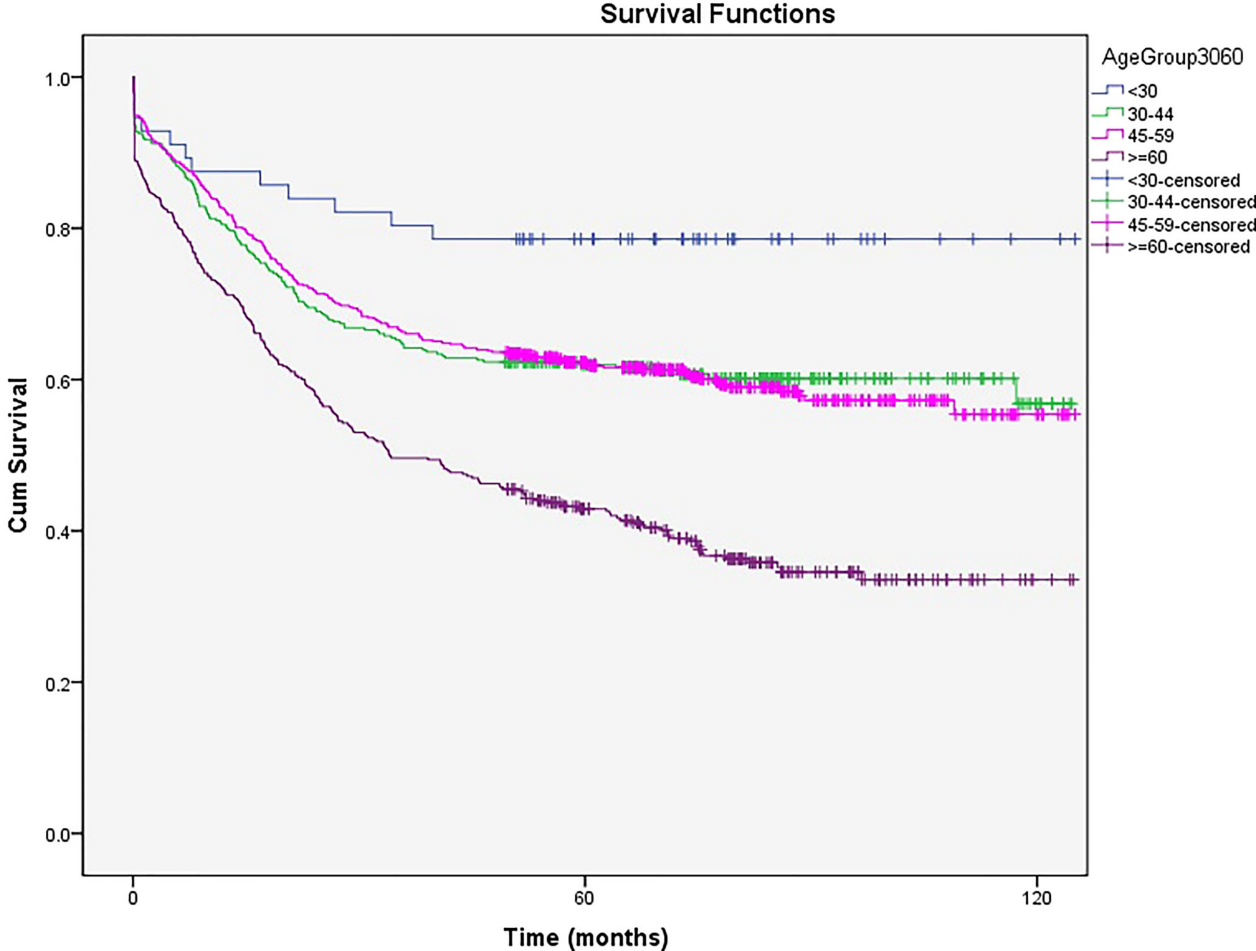
The 5- and 10-year survival rates of patients with cervical cancer based on aged groups.

After dividing the patients into four age groups, those younger than 30 had the best survival rate [5- and 10-year survival rates of 78% (77.92%- 78.08%)], and those aged 60 or older had the worst survival [5- and 10-year survival rates of 43% (42.94%- 43.06%) and 34% (33.92%- 34.08%), respectively, *P*<0.05]. Patients aged 30 to 44 and 45 to 59 had survival rates between the age groups mentioned above with no significant differences (5- and 10-year survival rates of 62% (61.94%-62.06%) and 57% (56.92%-57.08%) vs. 62% (61.96%-62.04%) and 56% (55.94%-56.06%), respectively) (**Figure 3**).

Based on the 13 regional centers mentioned above, the lowest 5- and 10-year survival rates were for Guilan in the north of Iran with 34% and 29%, respectively. Hamedan in the northwest of Iran had the best survival rates, with 5- and 10-year survival rates of 70% and 65%, respectively (**Table 2**).

**Table 2:** The 5- and 10-year survival rates and CI (95%) of cervical cancer based on regional centers.

Regional center	5-year (95%CI) (%)	10-year (95%CI) (%)
Hamedan	70 (69.86-70.14)	65 (64.84-65.16)
Fars	61 (60.94-61.06)	59 (58.94-59.06)
Tehran	60 (59.94-60.06)	52 (51.94-52.06)
Khorasan	58 (57.94-58.06)	55 (54.94-55.06)
Kerman	57 (56.90-60.10)	47 (46.88-47.12)
Alborz	57 (56.86-57.14)	54 (53.84-54.16)
Kermanshah	53 (52.86-53.14)	50 (49.86-50.14)
Qom	50 (49.86-50.14)	33 (32.82-33.18)
Azerbaijan	47 (46.92-47.08)	36 (35.92-36.08)
Khuzestan	46 (45.90-46.10)	42 (41.90-42.10)
Isfahan	45 (44.94-45.06)	39 (38.92-39.08)
Mazandaran	44 (43.92-44.08)	30 (29.88-30.12)
Guilan	34 (33.88-34.12)	29 (28.86-29.14)

CI, Confidence Interval.

**Figure 4:**
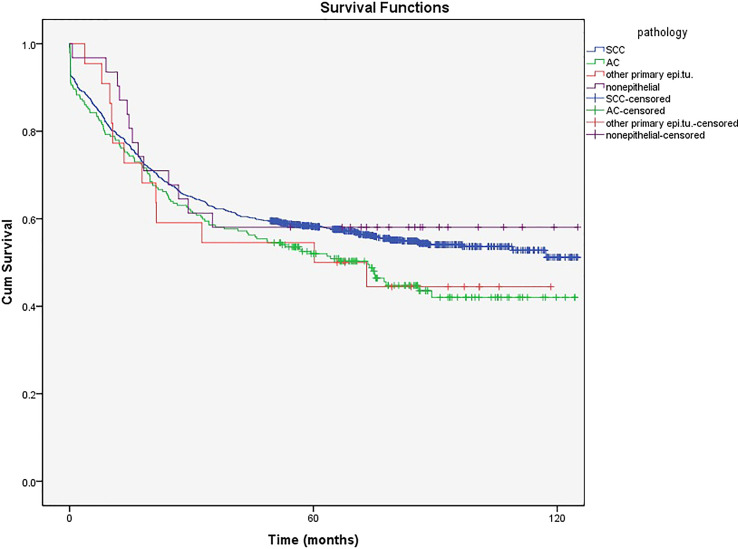
The 5- and 10-year survival rate of patients with cervical cancer based on pathology type.

**Figure 4** shows that the 5-year survival rate was significantly higher in patients with SCC, which is the most common pathological type diagnosed in the cervix (80%), than in those with AC (17.5%) (58% and 51% vs. 52% and 42%, respectively, *P*<0.001). The mean age in patients with SCC and AC was 51.28 and 57.37 years, respectively. The ICD-O-3 morphological codes and numbers of cases of each category are shown in Supplement (**Supplementary Table 1**).

The 5- and 10-year survival in non-epithelial tumors (58% (57.84%-58.16%) and 58% (57.82%-58.18%), respectively) and other primary epithelial (50% (49.78%-50.22%) and 44% (43.76%-44.24%), respectively) malignancies showed no significant difference between SCC and AC (**Figure 4**).

**Figure 5:**
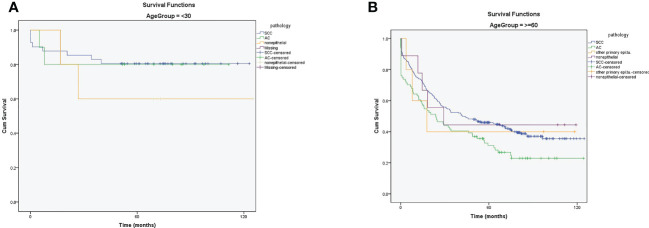
**(A)** The 5- and 10-year survival rates of patients with cervical cancer in ≤30 years based on pathology **(B)** The 5- and 10-year survival rates of patients with cervical cancer in ≥60 years based on pathology.

After adjusting age with pathology, there was no difference in the survival rate between SCC and AC in patients younger than 30 (**Figure 5A**). However, the 5- and 10-year survival rates were higher in SCC than in AC in patients aged older than 60 years (**Figure 5B**). (A table with numbers of cases and deaths by age group and histological types is shown in Supplement, **Supplementary Table 2**).

Cox proportional hazards regression analysis was performed on the age group, pathological group, and geographical segmentation variables. Age groups under 30 years, squamous cell carcinoma pathological type, and ​​Tehran as a geographical region were considered references, and the following results were obtained.

Survival was 2.13 times and 3.95 times worse in the age groups of 45-59 years and >60 years than in the age group > 30 years. Also, it showed no significant differences between the age groups 30-44 and <30 years.

The survival rate in the non-epithelial and other pathological groups was not significantly different from that in the squamous cell carcinoma group, but it was worse in the adenocarcinoma group than in the squamous group.

Regarding geographical segmentation, the survival rate was significantly worse in Mazandaran, Khuzestan, Isfahan, and Guilan than in Tehran. The worst survival rate was in the Guilan region (**Table 3**).

**Table 3:** Adjusted hazard ratios (95%CI) estimated for cox proportional hazards model for survival of cervical cancer.

Variables	Hazard Ratio (95% CI)
Age groups	
<30	1
30-44	1.763 (0.974-3.191)
45-59	2.133 (1.193-3.815) *
=>60	3.950 (2.215-7.045) *
Pathological types	
SCC	1
AC	1.434 (1.197- 1.718) *
Other	0.912 (0.499- 1.667)
Nonepithelial	0.833 (0.480- 1.447)
Regional Centers	
Tehran	1
Qom	1.478 (0.980- 2.228)
Mazandaran	1.532 (1.126-2.085) *
Khuzestan	1.509 (1.074-2.120) *
Khorasan	1.061 (0.795- 1.415)
Kermanshah	1.075 (0.687- 1.682)
Kerman	1.139 (0.787-1.647)
Isfahan	1.708 (1.315- 2.219) *
Hamedan	0.693 (0.371- 1.294)
Guilan	2.261 (1.595-3.207) *
Fars	1.217 (0.920-1.609)
Azerbaijan	1.568 (0.977- 2.516)
Alborz	1.122 (0.685- 1.835)

CI, Confidence Interval. *, significant.

## Discussion

Cervical cancer is the fourth leading cause of cancer death in women worldwide (1), highly associated with cultural and social issues. Although its strongest association is with HPV, different social behaviors can lead to varying outcomes (2). In a study by Khodakarami et al. in 2012, polygamous marriage, divorce, and the report of an individual’s husband who was not at home during the night for more than seven days per month were risk factor for positive HPV (6).

Cervical cancer is one of the most preventable cancers diagnosed by cellular changes in the Pap smear or HPV detection. Therefore, we expect CC incidence to reach the lowest possible rate with appropriate screening programs (12). Because of low CC incidence compared to western countries, CC screening in Iran, based on the Ministry of Health and Medical Education guidelines, starts at age 30 with 3 to 5- year intervals and ends at age 65 (6, 10).

The CC incidence is low in Iran, like in other Muslim countries in the Mediterranean region, (ASIR=1.9/10^5^) because of religious beliefs, social culture, and low screening, which is not among the 10^th^ most prevalent cancers (9, 10). However, CC is the most prevalent cancer in Sub-Saharan Africa and the second after breast cancer in North Africa (13). The CC cases increased steadily from March 10, 2008 to March 9, 2014 in Iran, parallel to findings in the Switzerland, Norway, and other European countries (12, 14). This increasing trend can be due to the detection of disease with screening, cultural deviation, inappropriate social behaviors (starting sex in younger ages out of the family system) or HPV prevalence (13).

According to **Figure 1**, the 45 to 54 years age group had the highest incidence rate, which is one decade later than in western countries and as the same as the situation of Asian American patients living in the United States (15). The peak age of CC diagnosis was lower in some western countries such as Estonia, Russia, and Scandinavia (40-49 years), most European countries (15-44 years), and the SEER study (35-44 years) than in west Asia and Iran (1, 13, 81–20). The mean age of CC incidence was higher in Korea than in West Asia and Iran (55-64 years) (16). As mentioned above, most Muslim women living in Asia and Iran restrain from having sexual intercourse before marriage and limit their sexual activity to only one partner after marriage. Hence, these countries have lower CC incidence and higher mean age of patients than western countries (2, 6, 9). The incidence rate of HPV infection, the most critical risk factor for CC worldwide, is lower at younger ages (2).

The mean age of CC patients in Iran was 51.91 years, which is higher than in several European countries (44.7 years) and the United States (50 years), but similar to that in a previous study (53.4 years) (15–19).

The mean ages of SCC and AC in Iran were 51.28 and 57.37, respectively, differing from those in Poland and Greater Amsterdam, in which the mean age of SCC was higher than that of AC (47 years compared to 44 years) (19, 20). One of the most important reasons for the higher mean age of CC in Iran than in western countries is their difference in the age population pyramid, as the highest population aggregation in Iran ranges from 45 to 54 years (21). Also it can be due to differences in the age-specific distribution of CC. The other difference is in cultural and religious beliefs, as Iranian women usually experience their first sexual practice after marriage and have only one partner at a time (2, 6, 9).

The 5- and 10-year survival rates of patients with CC in Iran from 2008 to 2014 were 58% and 50%, respectively. These rates are lower than those reported in several European countries, South America, South-East Asia, the United States, and Russia, with the 5-year survival rate ranging from 60% to 70% (1, 12, 22–24).

One of the reasons for the better survival rates in European countries is the availability of a comprehensive and well-organized screening program since 1960 (12, 14). However, according to the EUROCARE-3 study, since there is no uniform health care system for the prevention and treatment of patients with CC in Europe, 5-year survival rates are different among European countries. For instance, the highest survival rates are found in Northern and Western European countries (Denmark, Finland, Iceland, Norway, Sweden, Wales, and Scotland), while the lowest survival rates are found in central Europe (Slovenia, Slovakia, Estonia, and Poland) (12). Based on SEER 2018, establishing Pap smear test screening in the United States has improved the 5-year survival rates up to 66.1% (17).

Asian Americans have higher survival rates than white women (5-year survival rate of 0.70 vs. 0.67, *P* = 0.033), which can be due to the different sociocultural characteristics of Asians in terms of sexual behavior and marriage rate compared with white American women. Asian Americans are likelier to live with spouses or partners than white women (15).

Age at the time of diagnosis is the most crucial prognostic survival factor in patients with CC. Subsequently, patients’ survival rate decreases with age (24). In this study, the 5- and 10-year survival rates of patients younger than 30 years of age were 78% for both, and the corresponding survival rates in 60-year-old and older patients were 43% and 34%, respectively. Also, the 5-year survival rate of patients aged 30 to 44 and 45 to 54 years was 62%.

In multivariate analysis, there was no difference in the survival rate between patients younger than 30 and those aged 30-45. However, the survival rate was two and four times worse in patients aged 45-60 years and those older than 60 years, respectively. Because of self-neglect in older ages, patients are diagnosed in late stages, and thus palliative management is done for them.

The EUROCARE-3 study revealed that the 5-year survival rate of 75- to 99-year-old patients was less than 30%, with even 56% in some countries, which shows the existence of different treatment standards and screening systems for older patients based on the economic status of countries. However, the survival rate of patients younger than 50 years extensively increased from 85% to 90% (12, 20, 25).

In our study, older patients (≥60 years) had a lower 5-year survival rate than those in European countries as no organized screening program is available for this population (26, 27). Thus, patients are diagnosed late and receive conservative therapies due to their comorbidities. This should be a warning sign to emphasize screening programs and revisiting high-risk patients.

Generally, SCC pathological type has a higher survival rate than AC in Iran. As mentioned earlier, age is one of the most important prognostic factors of patient survival, and since the mean age was lower in patients with SCC than in those with AC, different survival rates were expected (51.28 and 57.37 years, respectively). Besides, AC has a lower sensitivity to radiotherapy and treatment response failure than SCC (28).

Cytological screening for AC prevention is not usually effective. At the same time, premalignant lesions, as a subtype of SCC, are more detectable by clinical and cytological investigations, leading to a decreased incidence of cervical SCC in many countries, as well as a diagnosis in the early stages of the disease and increased survival compared to AC (26).

In other countries, survival rates also vary based on the pathological type of tumors. In Estonia and China, the survival rates were higher in patients with SCC than in those with AC, similar to our study (26, 28). However, in Sweden, no significant difference existed between these two pathologies regarding the survival rate (27). According to the EUROCARE-3 study in 2003, this difference is not remarkable in many European countries. However, survival is higher in younger patients (<55 years) with AC and older patients (>65 years) with SCC (12, 25).

In the current study, after adjusting for age and pathology, the survival rate of young patients (≤ 30 years) with CC was not statistically different from that in the AC and SCC groups. However, the 5- and 10-year survival rates were higher in patients with SCC than those with AC. These differences can be explained by the reasons mentioned above. Since the mean age of patients with AC is high, the survival rates are expected to be higher in older patients with SCC than in patients with AC.

Cervical cancer also depends on the patients’ place of residence. The 5-year survival rate in the 13 regional centers of Iran varied between 34% and 70%, which can be due to differences in cultural behaviors and therapeutic care (13). Concerning the 13 regional centers of Iran, Guilan in the north had the lowest 5-year survival rate (34%), while Hamedan in the northwest of Iran had the highest 5- and 10-year survival rates during the study period. In the EUROCARE-3 study, the survival rate was different in different across countries. Accordingly, the north and west European countries had higher survival rates than the rest of the European countries (67.6% and 63.7%, respectively) (12).

## Limitations and strengths

This research was derived from Iran’s national cancer registry system and published for the first time. In this study, the sample size was large. On the other hand, this study had some limitations. To determine the vital condition, we called the phone numbers of the patients registered in the cancer registration system. Some of the phone numbers had been changed or recorded incorrectly. Moreover, it was not possible to find all of the patients due to immigration and emigration. Also, we did not have access to death certificates with valid dates. The data on the cancer registration system did not include the clinical characteristic of patients, such as tumor stage and pathological grade. The qualities of data registration systems were different, so we had to spend much time for cleaning the databases.

## Conclusions

As a preventable disease, CC is related to biological factors and geographical and sociodemographic indices. In Iran, 5-year survival rate differs from 34% to 70% geographically. Cultural issues and behavioral factors are essential in Iran, and social factors mainly affect CC incidence and survival rate, explaining the higher age of CC incidence in Iran compared to European and African countries. Pathology and age affect survival rate, with a better survival rate in younger women. Effective screening based on cultural and sociodemographic issues is recommended.

## Data availability statement

The data analyzed in this study is subject to the following licenses/restrictions: The raw dataset analyzed in this study is not publicly available. It can be provided on reasonable request through the corresponding author. Requests to access these datasets should be directed to khayamzadeh@yahoo.com.

## Author contributions

Study concept and design: AA, MK, MA. Analysis and interpretation of data: MK, RS, AM. Drafting and revision the manuscript: AA, MK, MA, RS, AM. Supervision: MA. All authors contributed to the article and approved the submitted version.
