# Supplementary material for: Epidemiology and survival of cervical cancer in Iran based on national cancer registry data (2008-2014)
Source: Front Oncol. 2023 Apr 19;13:1132369. doi: 10.3389/fonc.2023.1132369 (PMC10156133; doi:10.3389/fonc.2023.1132369)
Supplement: Supplementary file 1 [file Table_1.docx]

| **ICD**  **Code** | **Description** | **Count** | **4 group categorization** |
| --- | --- | --- | --- |
| 8000 | NEOPLASM | 572 | SCC |
| 8001 | NEOPLASM | 1 | SCC |
| 8002 | NEOPLASM | 3 | SCC |
| 8004 | NEOPLASM | 3 | SCC |
| 8010 | CARCINOMA, NOS | 317 | SCC |
| 8012 | CARCINOMA, NOS | 3 | SCC |
| 8013 | CARCINOMA, NOS | 3 | SCC |
| 8015 | CARCINOMA, NOS | 7 | SCC |
| 8020 | CARCINOMA, UNDIFF., NOS | 32 | SCC |
| 8031 | GIANT & SPINDLE CELL CARCINOMA | 1 | SCC |
| 8033 | GIANT & SPINDLE CELL CARCINOMA | 1 | SCC |
| 8041 | NON-SMALL CELL CARCINOMA, NOS | 6 | SCC |
| 8050 | PAPILLARY CARCINOMA, NOS | 12 | SCC |
| 8051 | PAPILLARY CARCINOMA, NOS | 1 | SCC |
| 8052 | PAPILLARY CARCINOMA, NOS | 15 | SCC |
| 8070 | SQUAMOUS CELL CARCINOMA, NOS | 1698 | SCC |
| 8071 | SQUAMOUS CELL CARCINOMA, NOS | 377 | SCC |
| 8072 | SQUAMOUS CELL CARCINOMA, NOS | 841 | SCC |
| 8073 | SQUAMOUS CELL CARCINOMA, NOS | 40 | SCC |
| 8074 | SQUAMOUS CELL CARCINOMA, NOS | 2 | SCC |
| 8075 | SQUAMOUS CELL CARCINOMA, NOS | 1 | SCC |
| 8076 | SQUAMOUS CELL CARCINOMA, NOS | 52 | SCC |
| 8077 | SQUAMOUS CELL CARCINOMA, NOS | 315 | SCC |
| 8082 | LYMPHOEPITHELIAL CARCINOMA | 1 | SCC |
| 8083 | LYMPHOEPITHELIAL CARCINOMA | 12 | SCC |
| 8084 | LYMPHOEPITHELIAL CARCINOMA | 1 | SCC |
| 8090 | BASAL CELL CARCINOMA, NOS | 5 | SCC |
| 8098 | BASAL CELL CARCINOMA, NOS | 2 | SCC |
| 8120 | TRANSITIONAL CELL CARCINOMA, NOS | 2 | SCC |
| 8123 | TRANSITIONAL CELL CARCINOMA, NOS | 1 | SCC |
| 8130 | PAPILLARY TRANS. CELL CARCINOMA | 4 | SCC |
| 8140 | ADENOCARCINOMA, NOS | 494 | AC |
| 8144 | ADENOCARCINOMA, NOS | 3 | AC |
| 8148 | ADENOCARCINOMA, NOS | 5 | AC |
| 8200 | ADENOID CYSTIC & CRIBRIFORM CA. | 5 | AC |
| 8210 | ADENOCA. IN ADENOMA. POLYP | 1 | AC |
| **8246** | **CARCINOID TUMOR, MALIGNANT** | 7 | other epithel. |
| 8260 | PAPILLARY ADENOCARCINOMA, NOS | 32 | AC |
| 8262 | PAPILLARY ADENOCARCINOMA, NOS | 5 | AC |
| 8263 | PAPILLARY ADENOCARCINOMA, NOS | 1 | AC |
| 8310 | CLEAR CELL ADENOCARCINOMA, NOS | 47 | AC |
| 8320 | GRANULAR CELL CARCINOMA | 1 | non- epithel. |
| **8380** | **ENDOMETRIOID ADENOCARCINOMA** | 88 | other epithel. |
| **8382** | **ENDOMETRIOID ADENOCARCINOMA** | 2 | other epithel. |
| **8384** | **ENDOMETRIOID ADENOCARCINOMA** | 26 | other epithel. |

| 8440 | CYSTADENOCARCINOMA, NOS | 1 | AC |
| --- | --- | --- | --- |
| 8441 | CYSTADENOCARCINOMA, NOS | 9 | AC |
| 8460 | PAPILLARY SEROUS CYSTADENOCA | 9 | AC |
| 8461 | PAPILLARY SEROUS CYSTADENOCA | 1 | AC |
| 8480 | MUCINOUS ADENOCARCINOMA | 22 | AC |
| 8481 | MUCINOUS ADENOCARCINOMA | 5 | AC |
| 8482 | MUCINOUS ADENOCARCINOMA | 7 | AC |
| 8490 | SIGNET RING CELL CARCINOMA | 3 | AC |
| 8500 | DUCT CARCINOMA | 3 | AC |
| **8560** | **ADENOSQUAMOUS CARCINOMA** | 61 | other epithel. |
| 8570 | ADENOCA. WITH METAPLASIA | 12 | AC |
| 8575 | ADENOCA. WITH METAPLASIA | 1 | AC |
| 8670 | LIPID CELL TUMOR, MAL. | 2 | non- epithel. |
| **8720** | **NEVI & MELANOMAS** | 4 | other epithel. |
| 8800 | SARCOMA, NOS | 7 | non- epithel. |
| 8801 | SARCOMA, NOS | 3 | non- epithel. |
| 8806 | SARCOMA, NOS | 2 | non- epithel. |
| 8814 | FIBROMATOUS NEOPLASMS | 1 | non- epithel. |
| 8890 | MYOMATOUS NEOPLASMS | 30 | non- epithel. |
| 8900 | RHABDOMYOSARCOMA, NOS | 3 | non- epithel. |
| 8910 | EMBRYONAL RHABDOMYOSARCOMA | 10 | non- epithel. |
| 8930 | STROMAL SARCOMA | 4 | non- epithel. |
| 8933 | STROMAL SARCOMA | 11 | non- epithel. |
| 8935 | STROMAL SARCOMA | 1 | non- epithel. |
| 8940 | MIXED TUMOR, MALIGNANT, NOS | 2 | other epithel. |
| 8950 | MULLERIAN MIXED TUMOR | 4 | other epithel. |
| 8951 | MULLERIAN MIXED TUMOR | 1 | other epithel. |
| 8980 | CARCINOSARCOMA, NOS | 12 | SCC |
| 8991 | MESENCHYMOMA, MALIGNANT | 1 | non- epithel. |
| **9072** | **EMBRYONAL CARCINOMA, NOS** | 2 | other epithel. |
| 9540 | NEUROFIBROSARCOMA | 1 | non- epithel. |
| 9581 | GRANULAR CELL TUMOR | 1 | non- epithel. |
| 9590 | MALIGNANT LYMPHOMA, NOS | 4 | non- epithel. |
| 9591 | MALIGNANT LYMPHOMA, NOS | 1 | non- epithel. |
| 9650 | HODGKIN LYMPHOMA | 1 | non- epithel. |
| 9663 | HODGKIN LYMPHOMA, NOD. SCLER. | 2 | non- epithel. |
| 9670 | ML, SMALL B-CELL LYMPHOCYTIC | 1 | non- epithel. |
| 9675 | ML, SMALL B-CELL LYMPHOCYTIC | 1 | non- epithel. |
| 9680 | ML, LARGE B-CELL, DIFFUSE | 7 | non- epithel. |
| 9687 | ML, LARGE B-CELL, DIFFUSE | 1 | non- epithel. |
| 9714 | OTHER SPEC. NON-HODGKIN LYMPHOMA | 1 | non- epithel. |
| 9727 | PRECURS. CELL LYMPHOBLASTIC LYMPH. | 1 | non- epithel. |
