# Supplementary material for: Epidemiology and survival of cervical cancer in Iran based on national cancer registry data (2008-2014)
Source: Front Oncol. 2023 Apr 19;13:1132369. doi: 10.3389/fonc.2023.1132369 (PMC10156133; doi:10.3389/fonc.2023.1132369)
Supplement: Supplementary file 2 [file Table_2.docx]

| Status | Age Group | Pathology | | | | Total |
| --- | --- | --- | --- | --- | --- | --- |
|  |  | SCC | AC | other primary epi.tu. | nonepithelial |  |
| Dead | <30 | 12 | 3 | 0 | 3 | 18 |
|  | 30-44 | 201 | 18 | 2 | 3 | 224 |
|  | 45-59 | 361 | 71 | 12 | 9 | 453 |
|  | >=60 | 376 | 168 | 6 | 9 | 559 |
|  | Total | 950 | 260 | 20 | 24 | 1254 |
| Alive | <30 | 50 | 12 | 0 | 5 | 67 |
|  | 30-44 | 300 | 35 | 1 | 9 | 345 |
|  | 45-59 | 423 | 76 | 11 | 8 | 518 |
|  | >=60 | 194 | 36 | 3 | 6 | 239 |
|  | Total | 967 | 159 | 15 | 28 | 1169 |
| Total | <30 | 62 | 15 | 0 | 8 | 85 |
|  | 30-44 | 501 | 53 | 3 | 12 | 569 |
|  | 45-59 | 785 | 148 | 23 | 17 | 973 |
|  | >=60 | 570 | 201 | 9 | 15 | 795 |
|  | Total | 1917 | 419 | 35 | 52 | 2423 |
